# Microevolution Analysis of *Bacillus coahuilensis* Unveils Differences in Phosphorus Acquisition Strategies and Their Regulation

**DOI:** 10.3389/fmicb.2016.00058

**Published:** 2016-02-08

**Authors:** Zulema Gómez-Lunar, Ismael Hernández-González, María-Dolores Rodríguez-Torres, Valeria Souza, Gabriela Olmedo-Álvarez

**Affiliations:** ^1^Laboratorio de Biología Molecular y Ecología Microbiana, Departamento de Ingeniería Genética, Unidad Irapuato, Centro de Investigación y de Estudios Avanzados del Instituto Politécnico NacionalIrapuato, Mexico; ^2^Laboratorio de Evolución Molecular y Experimental, Departamento de Ecología Evolutiva, Instituto de Ecología, Universidad Nacional Autónoma de MéxicoMéxico City, Mexico

**Keywords:** microevolution, comparative genomics, intraspecific diversity, phenotypic-genotypic association, mobile genetic elements, phosphorus limitation, phosphonate transport, Kdo biosynthesis

## Abstract

Bacterial genomes undergo numerous events of gene losses and gains that generate genome variability among strains of the same species (microevolution). Our aim was to compare the genomes and relevant phenotypes of three *Bacillus coahuilensis* strains from two oligotrophic hydrological systems in the Cuatro Ciénegas Basin (México), to unveil the environmental challenges that this species cope with, and the microevolutionary differences in these genotypes. Since the strains were isolated from a low P environment, we placed emphasis on the search of different phosphorus acquisition strategies. The three *B. coahuilensis* strains exhibited similar numbers of coding DNA sequences, of which 82% (2,893) constituted the core genome, and 18% corresponded to accessory genes. Most of the genes in this last group were associated with mobile genetic elements (MGEs) or were annotated as hypothetical proteins. Ten percent of the pangenome consisted of strain-specific genes. Alignment of the three *B. coahuilensis* genomes indicated a high level of synteny and revealed the presence of several genomic islands. Unexpectedly, one of these islands contained genes that encode the 2-keto-3-deoxymannooctulosonic acid (Kdo) biosynthesis enzymes, a feature associated to cell walls of Gram-negative bacteria. Some microevolutionary changes were clearly associated with MGEs. Our analysis revealed inconsistencies between phenotype and genotype, which we suggest result from the impossibility to map regulatory features to genome analysis. Experimental results revealed variability in the types and numbers of auxotrophies between the strains that could not consistently be explained by *in silico* metabolic models. Several intraspecific differences in preferences for carbohydrate and phosphorus utilization were observed. Regarding phosphorus recycling, scavenging, and storage, variations were found between the three genomes. The three strains exhibited differences regarding alkaline phosphatase that revealed that in addition to gene gain and loss, regulation adjustment of gene expression also has contributed to the intraspecific diversity of *B. coahuilensis*.

## Introduction

Bacterial genomes are highly dynamic and continually undergo numerous events of gains and losses of genes, which contribute to variation in genome size, gene content, and order (Koonin and Wolf, 2008). These genome rearrangements provide genome plasticity and generate new genome variants within the same species (Radnedge et al., 2002; Dobrindt et al., 2004). Modifications of gene repertoire arise via different mechanisms. For example, bacterial genomes acquire material from other organisms by horizontal gene transfer (HGT). The HGT can result in the addition of a completely new gene, the replacement of an existing gene, or genetic redundancy if a homologous gene is already present in the recipient genome (xenolog genes; Mira et al., 2002; Abby and Daubin, 2007). Gene duplication is an additional mechanism, which results in the formation of paralog genes and increase in genome size (Mira et al., 2001). The mechanisms that result in gene loss are gene excision and formation of pseudogenes, which arise when mutations (i.e., point mutations, insertions, deletions) accumulate and result in function loss (Ochman and Davalos, 2006). After this, evolutionary forces allow genes to remain or to be lost, and variants occur over time and space that promote individual adaptation to the environment (Ochman, 2001; Ochman and Moran, 2001; Maughan et al., 2006). On the other hand, genome arrangements and nucleotide polymorphisms among strains also confer high genomic diversity upon prokaryotic species (Ochman and Davalos, 2006; Abby and Daubin, 2007).

Evolutionary changes that occur below the level of species are referred to as microevolution (Abby and Daubin, 2007). Comparative genomic studies have shown that even bacterial strains belonging to the same species exhibit considerable variation in gene content as a result of events of DNA gains and losses (Tettelin et al., 2008; Rouli et al., 2015). The pangenome concept arose from the comparison of genomes from a given study group to define the gene repertoire (Tettelin et al., 2008). The core genome concept defines the subgroup of genes shared between all compared genomes (Mira et al., 2010; Vernikos et al., 2015). Genes present in only one or two strains are considered part of the accessory genome, it may some times be located in genomic islands (GI), and the genetic material is usually derived from HGT. The accessory genome may represent a survival strategy when it provides sets of genes from other organisms that may allow the recipient to adapt to new environments (Cohan and Koeppel, 2008). In this sense, recent studies of a bacterial species genomes suggest that the strain-specific genes contribute to individual adaptation to specific environmental conditions (Math et al., 2012; Penn and Jensen, 2012; Tian et al., 2012).

An analysis of the genome of *Bacillus coahuilensis* m4-4 (Alcaraz et al., 2008), a strain isolated from a desiccation lagoon in the Churince system in the Cuatro Ciénegas Basin (CCB; Cerritos et al., 2008) revealed events of gene gain and loss. Particularly, two adaptations to an oligotrophic environment were found. The first one is the presence of genes that encode sulfolipid biosynthesis (*sqd1* and *sqdX*), which likely increase fitness by reducing phosphorus utilization. The second adaptation is the occurrence of a high number of ATP-binding cassette (ABC) transporter systems for amino acids and oligopeptides, that suggests a high dependency on nutrients synthetized by other organisms in the microbial community (Alcaraz et al., 2008). Another finding was that *B. coahuilensis* has one of the smallest genomes of the *Bacillus* genus (3.38 Mpb; Alcaraz et al., 2008). Only three strains of this species have been recovered in the recent years of extensive isolation of thousands of *Bacillus* sp. from the CCB hydrological systems, and thus only relatively small populations of *B. coahuilensis* are likely present in the CCB.

The CCB is located in the Chihuahua Desert (Coahuila, North Central México). The basin is surrounded by high mountains (>3,000 m altitude). Stratigraphic evidence supports the theory that this valley had a marine origin (Mckee et al., 1990). Exceptional biodiversity and large numbers of endemic plant and animal species are characteristic of this area (Minckley, 1969; Johnson, 2005; Carson and Dowling, 2006). Within the basin, a large number of highly diverse springs (>300), spring-fed streams and evaporation ponds form an inverse archipelago, in which aquatic systems are separated by sparse desert vegetation, and salty soils (Souza et al., 2008). A high aquatic microbial diversity has also been described, and almost 50% of the bacteria isolated from CCB ponds were related to marine bacteria (Souza et al., 2006; Cerritos et al., 2011). The abundance of taxa related to marine bacteria leads to the hypothesis that some portion of the biota and water of CCB may have been derived from microbes and water entrapped in the Mesozoic strata; which have been released recently during ongoing subsurface karstification (Souza et al., 2006). An outstanding characteristic of the CCB is the extremely low nutrient levels in the hydrological systems (Souza et al., 2008). The phosphorus levels, in particular, appear to be low in the CCB (i.e., ∼0.60 μMol of total P; Elser et al., 2005). Bacteria may have several strategies to survive in low phosphorus environments, such as the replacement of membrane phospholipids for sulfolipids, small genome size, and enzymes to scavenge for alternative sources of phosphorus, like phosphonates (Dufresne et al., 2005; Dyhrman et al., 2006; Van Mooy et al., 2009; Villarreal-Chiu et al., 2012). The particular geological characteristics and low nutrient availability of CCB make it a suitable location to describe intraspecific genomic diversity in species that have survived in an ancient oligotrophic environment.

The aim of this study was to analyze the genomic and phenotypic variation between three *B. coahuilensis* strains to describe their genome plasticity. How many differences would there be among strains that are free living, but have a small genome and are exposed to similar environmental conditions? We sequenced, assembled, and annotated the genomes of *B. coahuilensis* m2-6, and p1.1.43; and compared them with the already published *B. coahuilensis* m4-4 genome. Since the strains were isolated from the same low P environment, we placed emphasis on the search of different strategies for phosphorus metabolism. Experimental assays revealed genotype-phenotype inconsistencies, probably the result of regulatory differences. We describe microevolutionary changes of the *B. coahuilensis* strains globally and, in particular, differences in phosphorus acquisition strategies among the strains.

## Materials and Methods

### Sample Locations and Culture Media

Three *B. coahuilensis* strains were isolated from water samples from different hydrological systems in the CCB. *B. coahuilensis* m4-4, was isolated from a desiccation lagoon (Churince system) with a C:N:P ratio of 50:7.5:1, and is located at the west side of the valley (26° 50.830′N, 102° 09.335′W; Cerritos et al., 2008). In this side of the valley the dominant soils are gypsisols (IUSS Working Group WRB, 2014). The strain *B. coahuilensis* m2-6 was also isolated from the Churince system (26° 51.199′N, 102° 09.009′W; Cerritos et al., 2011). Finally, the strain *B. coahuilensis* p1.1.43 was isolated from within the east side of the basin in the spring pool Poza Azul I (26°49.642′N, 102°01.458′E) with a C:N:P ratio of 51:1.8:1 (Cerritos et al., 2011). In this side of the valley the dominant soils are calcisols (IUSS Working Group WRB, 2014). Marine Medium (MM) was used for general culture of all afore mentioned strains [5% casein hydrolysate, 1% yeast extract, 85.55 mM NaCl, 7.04 mM Na_2_SO_4_, 3.60 mM CaCl, 2.68 mM KCl, 0.94 mM Na_2_CO_3_, 0.29 mM C_6_H_5_FeO_7_, 40 mM MgSO_4_, 0.67 mM KBr, 0.35 mM H_3_BO_3_, 0.57 mM NaF, 0.19 mM NO_3_NH_4_, and 0.56 mM Na_2_HPO_4_].

### Comparative Genomics

#### Genome Sequencing, Assembly, and Annotation

Cultures of the *B. coahuilensis* strains were grown in liquid MM overnight at 37°C. Genomic DNA was obtained using the modified rapid method for the isolation of genomic DNA (Hoffman and Winston, 1987). Genomic DNA was quantified using a NanoDrop spectrophotometer (ND-1000, Thermo Fisher Scientific, Wilmington, DE, USA). Two sequencing technologies were used to sequence the strain genomes: shotgun libraries sequenced with the GS-FLX Titanium^®^ pyrosequencer (454 Life Sciences, Roche, Branford, CT, USA), located at the Cinvestav–Langebio (Irapuato, México), and a mate pair libraries sequencing analysis (5-kb inserts) with an Illumina^®^ genome analyzer (Illumina, San Diego, CA, USA), located at the Instituto de Biotecnología-UNAM (Cuernavaca, México). Pyrosequence reads were assembled using a Celera assembler v8.2 (Celera Genomics, Rockville, MD, USA; Myers et al., 2000).

Contigs were ordered and oriented by mapping the Illumina mate-pair reads to the Celera contigs using the alignment program BOWTIE v1.1.2 (Langmead et al., 2009). To optimize genome scaffolding, we only used the illumina reads that mapped once in each contig. The *B. coahuilensis* m4-4 genome was re-sequenced, using the mate-pair library sequencing with 5-kb inserts and the Illumina analyzer. The Illumina mate-pair reads were also mapped to the previously reported contigs (Alcaraz et al., 2008). The quality assessment for genome assemblies was done using QUAST and CheckM (Gurevich et al., 2013; Parks et al., 2015). The complete genomic sequences of *B. coahuilensis* strains m2-6 and p1.1.43 have been deposited in GenBank under the accession numbers LDYF00000000.1 and LDYG0000000.1, respectively. The average nucleotide identity (ANI) was calculated according to Goris et al. (2007). The open reading frames (ORFs) were predicted and annotated using RAST v2.0 (Rapid Annotation using Subsystem Technology; Aziz et al., 2008). The genome-scale metabolic models were obtained using Model SEED v1.0 (Overbeek et al., 2005). The results for the metabolic models of amino acid biosynthesis, carbohydrate utilization, ability to swarm and swim, and phosphate metabolism, were compared with the results obtained from experimental data.

#### Determination of Accessory Genome and Genomic Islands

*Bacillus coahuilensis* core genome and accessory genes were identified searching for orthologous genes, according to a previously described methodology (Moreno-Hagelsieb and Latimer, 2008). The genome alignment was performed and visualized using the Mauve software tool v2.4 (Darling et al., 2004). GI were identified using the Genomic Island Suite of Tools GIST v1.0 (Hasan et al., 2012) and determination of GC% in these genomic regions. Identification of mobile genetic elements (MGE) was performed using PHAST web server for the phage sequences and ISsaga v1.0 for the insertion sequences (ISs; Varani et al., 2011; Zhou et al., 2011).

To search for genes encode phosphonate metabolism, we built profile hidden Markov models using the software HMMER v3.1 (Finn et al., 2011). We built profiles based on protein alignments of sequences obtained from the Genbank^1^ for each protein, including phosphonopyruvate hydrolase (PalA), phosphonoacetate hydrolase (PhnA), transcriptional regulator (PhnW), repressor protein (PhnF), phosphonate lyase (PhnGHM), phosphonate transporters (PhnCDE1E2), and phosphonoaldehyde dehydrogenase (PhnXY).

#### Inferring Horizontal Gene Transfer (HGT) of Phosphonate Transporter and Proteins of 2-Keto-3-Deoxymannooctulosonic Acid (Kdo) Biosynthesis

To analyze whether the genes encoding the phosphonate transporter and the Kdo biosynthesis proteins had been transferred by HGT into *B. coahuilensis* m2-6 genome, we first searched for genomic signatures of: (a) significant difference in GC content for the genome segments that can be an indication of foreign DNA, and (b) analysis of the genomic context, searching for flanking repeat sequences and presence of nearby integrases or transposases genes that could suggest a non-native region (Ravenhall et al., 2015). We also inferred HGT by analyzing the phylogeny where we compared gene tree with their associated species tree to search for unusual distribution of the genes that probably are from HGT (Ravenhall et al., 2015). This was the case for the phosphonate transporter gene, for which we did a phylogenetic reconstruction. Multiple alignment of the Phn amino acid sequences of several species of the class Bacilli (PhnC, PhnD, PhnE1, and PhnE2) were assembled using Muscle v3.8.31 (Edgar, 2004). The alignments were further concatenated and used in the phylogenetic analyses, which in turn were performed using maximum likelihood estimation software PhyML v3.0 (Guindon et al., 2010). The LG substitution model was supported by the test results (–lnL = 7221.82; Abascal et al., 2005). *Clostridium ultunense* Phn transporter sequences were used as outgroup. Bootstrap values were calculated after 100 pseudo-replicates to estimate nodal support. We used the same phylogenetic inference analysis strategy to determine the KdsB protein (3-deoxy-manno-octulosanate cytidylyltransferase) associated with Kdo biosynthesis. The JTT substitution model was supported by the test results (–lnL = 9752.74). The *Arabidopsis thaliana* KdsB sequence was used as outgroup. The species tree was based on the phylogenetic reconstruction of the 16S rRNA gene. The GTR substitution model was supported by the test results (–lnL = 11838.64). The *Clostridium ultunense* 16S rRNA sequence was used as outgroup.

### Experimental Assays for Phenotypic Diversity

#### Amino Acid Requirements

The three *B. coahuilensis* strains were plated on modified phosphate defined medium (PDM) and incubated at 37°C for 48 h to determine amino acid requirements (Muller et al., 1997). The PDM medium contained 50 mM Tris, pH 7.5, 85.55 mM NaCl, 3.6 mM CaCl_2_, 3.98 mM MgSO_4_, 0.19 mM NH_4_NO_3_, 7.71 mM Na_3_C_6_H_5_O_7_, 1.58 mM MnCl_2_, 0.001 mM ZnCl_2_, 1.66 mM FeCl_3_, 1.39 mM KH_2_PO_4_, and 49.95 mM glucose. We added for each amino acid: 50 μg/mL of D-alanine, L-arginine, L-asparagine, L-glutamine, L-glycine, L-histidine, L-isoleucine, L-leucine, L-lysine, L-methionine, L-phenylalanine, L-proline, L-serine, L-threonine, and L-valine; 500 μg/mL of L-aspartic acid, and L-glutamic acid; 40 μg/mL of L-cysteine; 20 μg/mL of L-tryptophan, and L-tyrosine (Harwood and Cutting, 1990). Separate plates were inoculated to perform three biological replicates.

#### Differences in Carbon Source Utilization

The *B. coahuilensis* strains were plated on Biolog Universal Agar (BUG^TM^ Agar; Biolog, Inc., Hayward, CA, USA). MM salts were added to improve strain growth and thioglycolate was added to decrease bacterial capsule biosynthesis. The plates were incubated at 30°C for 24 h. Subsequent steps were performed according to the manufacturer’s instructions. Three replicate of each strain were prepared. The absorbance values were normalized by calculating dual wavelength data (DWD) according to the manufacturer’s formula.

#### Swarming and Swimming

The ability to move to search for nutrients or respond to favorable or unfavorable conditions is another important phenotypic characteristic of microorganisms living in oligotrophic environments. For swarming assays, plates (9 cm) containing 25 mL MM (0.6% agar) were prepared 1 h before inoculation, and dried with the lids open for 15 min in a laminar-flow hood. The plates were centrally inoculated with 2 × 10^7^ cells in 10 μL, the lid was left open for another 10 min, and the plates were then incubated at 37°C for 24 h. Swimming/motility assays were performed on MM with 0.3% agar by inoculating cells in a 10 μL (at the center of each plate), and incubating at 37°C for 24 h (Kearns and Losick, 2003).

#### Differences in Phosphorus Source Utilization

To determine growth when bacteria were exposed to different phosphorus (P) sources, the three strains were plated on PDM solid medium at 37°C for 48 h. They were then re-streaked onto PDM media containing different phosphorus sources 1.4 mM KH_2_PO_4,_ 0.1 mM Ca_3_(PO_4_)_2,_ 0.1 mM 5-bromo-4-chloro-3-indolyl phosphate (X-P), 0.1 mM Na_2_HPO_3_, 0.1 mM 2-aminoethylphosphonic acid, 0.1 mM phosphonoacetaldehyde, 0.1% DNA, and 0.1% RNA. After 48 h of incubation, the strains were then streaked again on the identical medium and the same phosphorus source to confirm the growth phenotypes. Separate plates were inoculated with three biological replicates.

#### Alkaline Phosphatase Activity

Alkaline phosphatase activity was assayed at high and low concentrations of phosphorus. The high PDM (HPDM) contained 1.4 mM KH_2_PO_4_. No KH_2_PO_4_ was added to the low PDM (LPDM), so the bacteria were exposed only to the trace phosphorus present in the reagents used for the preparation of the medium. A molybdate colorimetric method after ascorbic acid reduction was used to determine the final concentration of phosphorus in the media (147.1 mg P mL^-1^ HPDM and 3.3 mg P mL^-1^ LPDM). Strains were inoculated in liquid MM at 37°C for 24 h. Strains were inoculated on LPDM or HPDM solid medium containing 40 μg/mL 5-Bromo-4-chloro-3-indolyl phosphate (X-Pi) as an indicator of alkaline phosphatase activity (Metcalf and Wanner, 1991). Separate plates were inoculated with three biological replicates.

## Results

### Characteristics of the *B. coahuilensis* m2-6 and *B. coahuilensis* p1.1.43 Genomes

The *B. coahuilensis* m2-6 and *B. coahuilensis* p1.1.43 genomes were sequenced using 454 technology at 62x and 64x coverage, respectively. The assembly using 454 and Illumina data resulted in 71 contigs for *B. coahuilensis* m2-6, and 59 contigs for *B. coahuilensis* p1.1.43. The results obtained and comparisons with the results of *B. coahuilensis* m4-4 genome (accession number NZ_ABFU00000000.1; Alcaraz et al., 2008) are presented in **Table 1**. The results of the quality assessment for genome assemblies indicated that the three genomes could be considered near-complete with low contamination (**Table 1**). The ANI value between *B. coahuilensis* m4-4 and *B. coahuilensis* m2-6 was the highest (99.37%). The ANI value between *B. coahuilensis* m2-6 and *B. coahuilensis* p1.1.43 was 98.93%. And for *B. coahuilensis* m4-4 and *B. coahuilensis* p1.1.43, the ANI was the lowest with 98.88%. All the ANI values are in agreement with those reported for strains of the same species. The results indicated that the *B. coahuilensis* strains had small differences in Coding DNA Sequences (CDS) number (**Table 1**). Furthermore, there were minor differences in the percent values for guanine–cytosine (GC) content. Strain *B. coahuilensis* m2-6 had the highest percent GC value (37.97%), followed by strain p1.1.43 (37.96%) and strain m4-4 (37.92 %). The results indicated that there were differences in genome size. *B. coahuilensis* p1.1.43 had the largest (3.40 Mbp), and *B. coahuilensis* m2-6 had the smallest (3.21 Mbp) genome.

**Table 1:** General characteristics of the *Bacillus coahuilensis* genomes.

	*Bacillus coahuilensis* strains		
Characteristics	m4-4	m2-6	p1.1.43
**Source**	**Churince**	**Churince**	**Pozas Azules I**
Genome size (Mbp)	3.34	3.21	3.40
G + C%	37.92	37.97	37.96
Coverage	35x	62x	64x
# contigs	78	71	59
N50(bp)	67194	75938	88256
L50	14	15	12
Mean contigs length (bp)	42906	45287	57766
Largest contig length (bp)	256246	192207	272931
Largest scaffold length (bp)	653377	1022029	1719176
# scaffolds	28	9	7
Completeness (%)^∗^	98.65	98.36	99.34
Contamination (%)^∗^	1.99	0.88	0.22
Coding DNA sequences (CDS)	3556	3500	3530
rRNAs	21	16	9
tRNAs	76	78	75
Reference	Alcaraz et al., 2008	This study	This study

*^∗^Completeness and contamination percentages were calculated using CheckM*.

### Pangenome and Mobile Genetic Element Diversity

**FIGURE 1:**
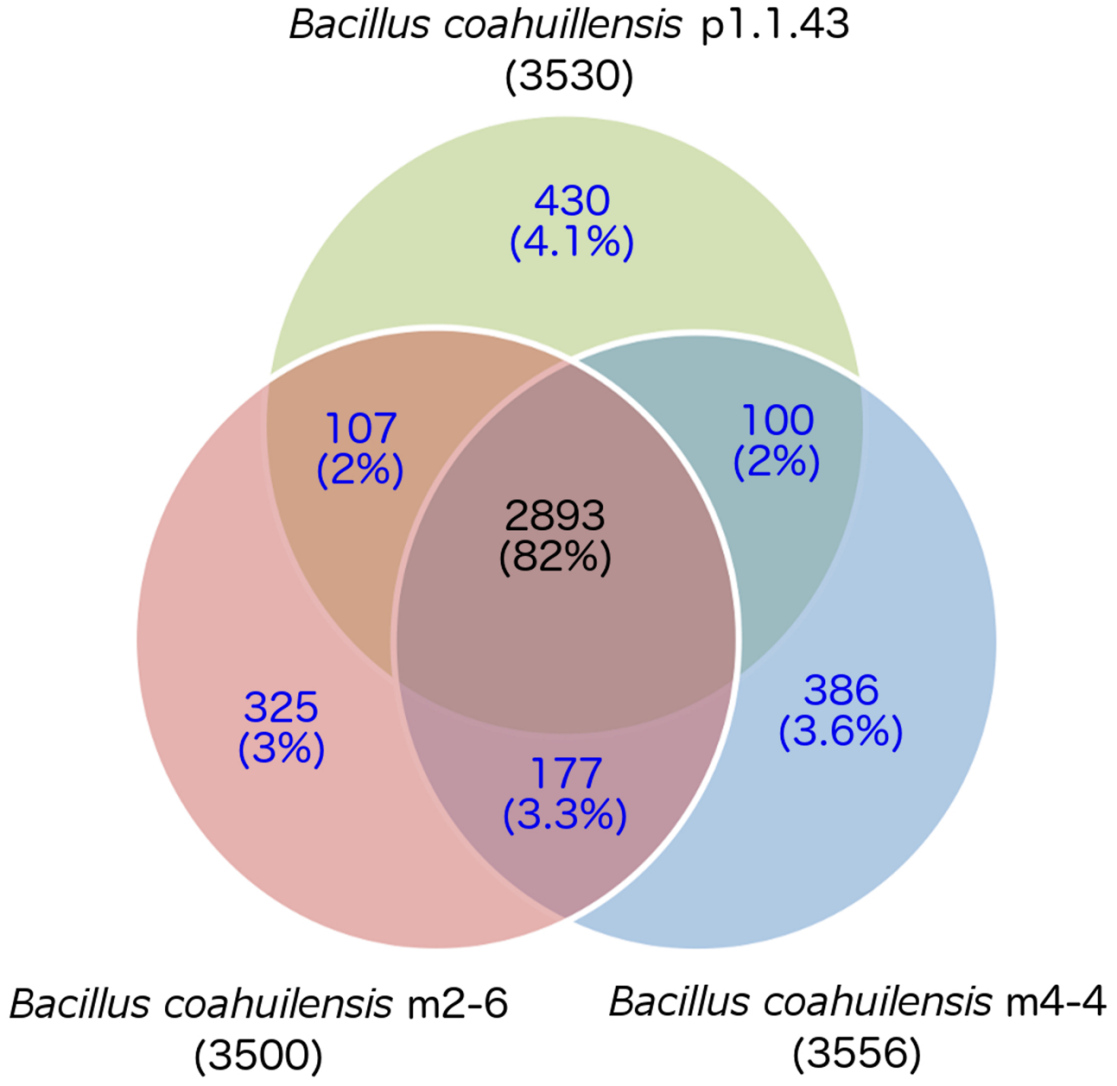
**Core and accessory genomes of three *Bacillus coahuilensis* strains (m4-4, m2-6, and p1.1.43)**. Ortholog identification was performed using the bidirectional best hit (BBH) approach with mutual coverage or a shared length of 60% and an e-value ≥10^-6^. The core genome is indicated by the numbers in black font. Accessory gene numbers are in blue font. The number of predicted genes is shown in parenthesis for each genome.

**FIGURE 2:**
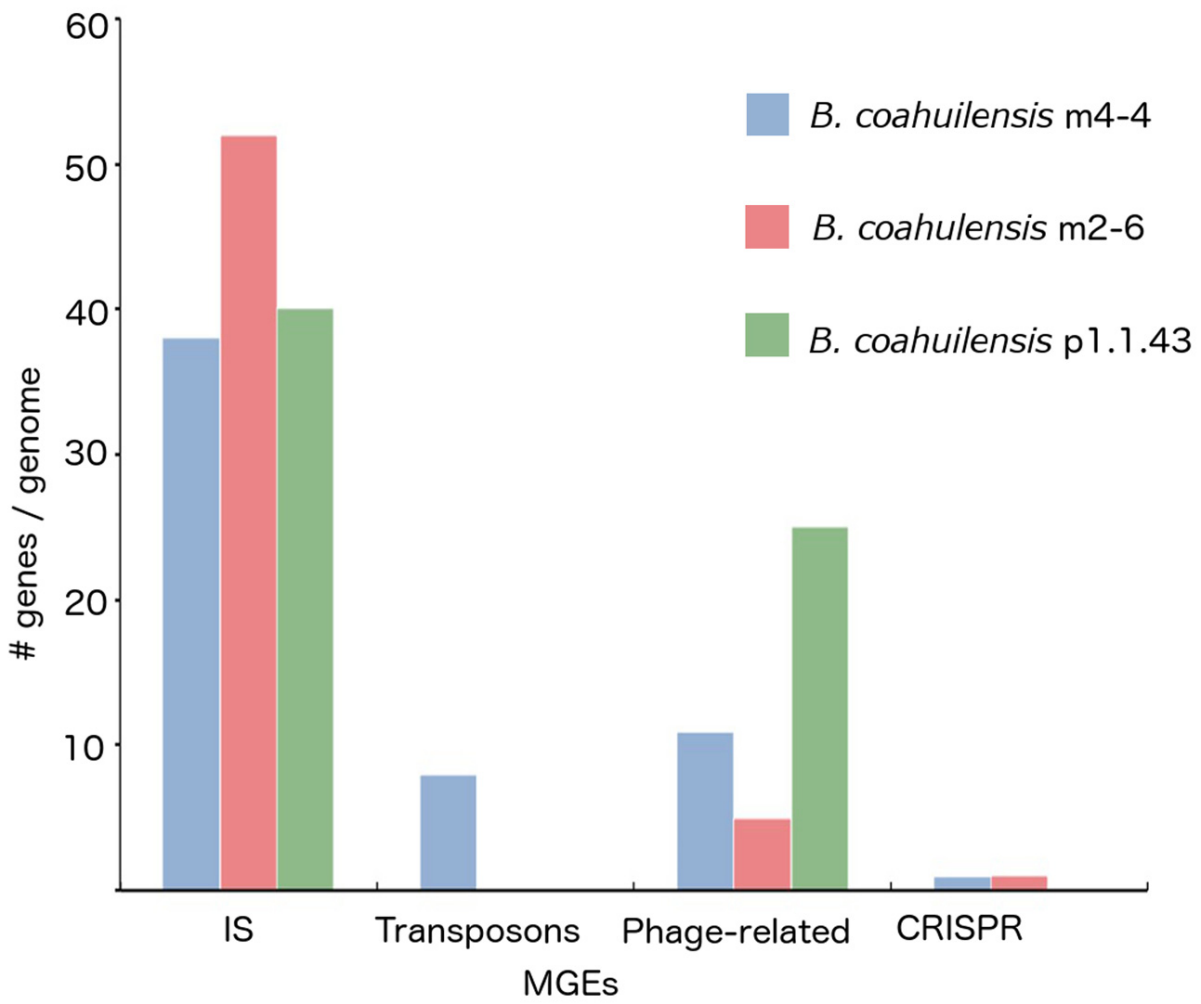
**Mobile genetic elements (MGEs) identified in the *B. coahuilensis* genomes (strains m4-4, m2-6, and p1.1.43)**. Axis Y indicates the number of genes related to MGEs per genome, axis X indicates different MGEs coding in the genomes (IS, transposons, phage-related genes, and CRISPR).

The core genome shared between the *B. coahuilensis* strains was determined using previously published methods (Moreno-Hagelsieb and Latimer, 2008). Comparison of the orthologous genes revealed that the pangenome consisted of 4,416 genes, and the core genome consisted of 2,893 orthologous genes (82%). The accessory genome was 18% of the pangenome. Strain p1.1.43 had the largest number of strain-specific genes (4.1%), followed by strain m4-4 (3.6%) and m2-6 (3.0%; **Figure 1**). *B. coahuilensis* m4-4 and m2-6 (both isolated from the Churince pond) shared more genes between them than with strain p1.1.43 (isolated from Pozas Azules; **Figure 1**). The search for MGE revealed that IS and phage-related genes were present in all three genomes. The m4-4 and m2-6 genomes had clustered regularly interspaced short palindromic repeat (CRISPR) genes, in contrast; we did not find CRISPR in the p1.1.43 genome. The *B. coahuilensis* m2-6 genome had a higher number of ORFs related to ISs (52) compared with the other two genomes. The *B. coahuilensis* m4-4 is the only one genome that possesses genes coding for Tn7 (8). The p1.1.43 genome had more phage-related genes (25) than the m2-6 (5) and m4-4 genomes (11) (**Figure 2**). We also investigated what was encoded in the accessory genes and, in particular, if there were MGEs that could explain the differences in the *B. coahuilensis* genomic repertoire. Some MGEs were shared by the m4-4 and the m2-6 strains (e.g., CRISPR, ISs). Strains m4-4 and p1.143 shared phage-related genes. As previously described in **Figure 1**, strain p1.1.43 had more strain-specific genes compared with the other two genomes of which 5.3% were MGEs. For example, some of the MGEs were phage-related genes, and some were ISs (IS*660*, IS*1182*, IS*Shvi3*, IS*Bsp1*, IS*Arsp6*, Tn*Shfr1*, IS*Xc4*, and IS*Swi1*). Approximately 5% of the strain-specific genes present in strain m2-6 were MGEs; the remainder were mostly ISs (IS*Bspe1*, IS*Bsp3*, IS*Cth6*, IS*Sep2*, IS*Enfa110*, IS*Pa38*, and IS*Acsp1*). We also found that 8.79% of the strain-specific genes present in strain m4-4 were phage-related genes, ISs (IS*1326*, IS*Sm4*, IS*Bce19*, IS*Bse1*, IS*Baps1*, and IS*Cth6*), and that transposons were also present.

### Synteny and Identification of Genomic Islands

**FIGURE 3:**
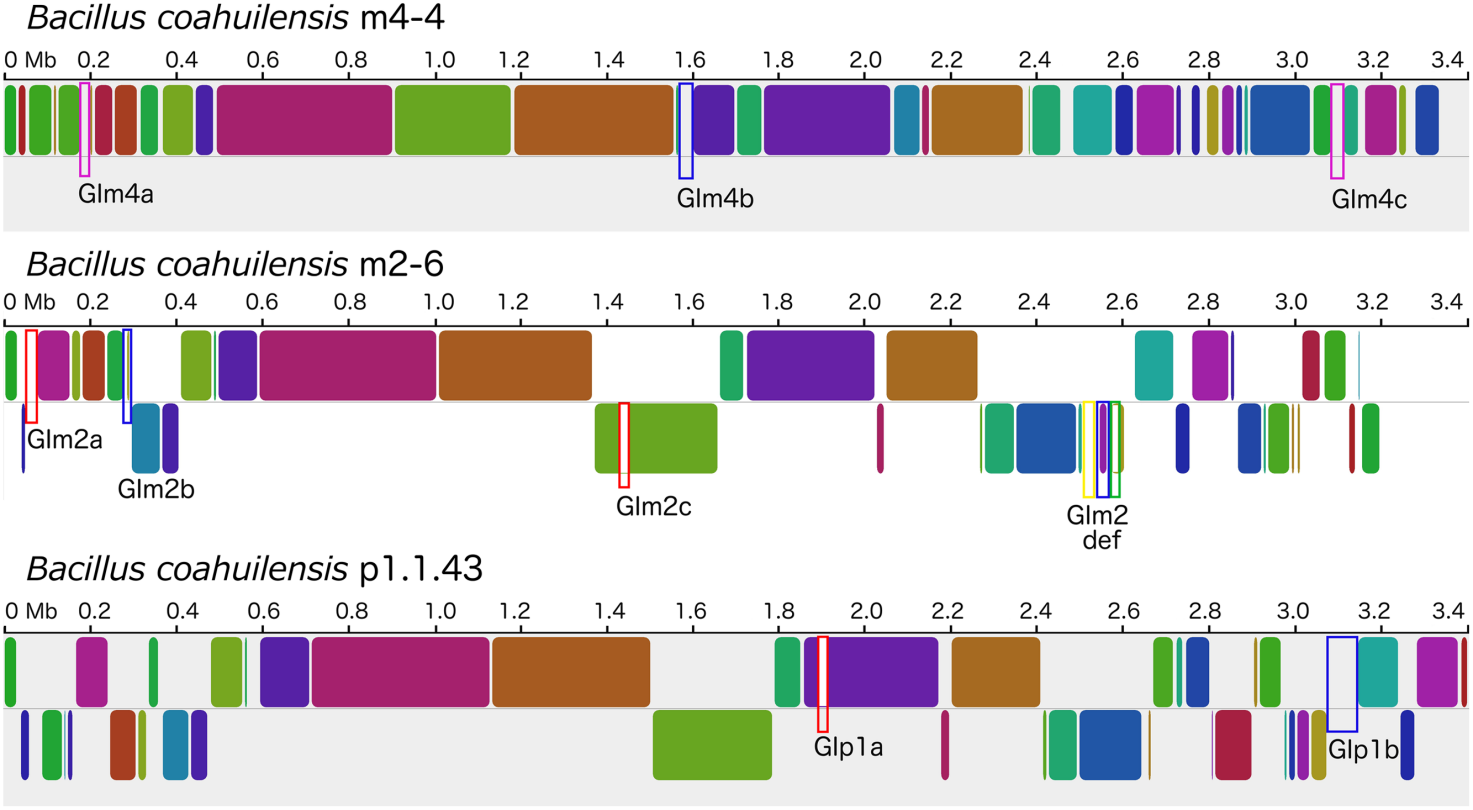
**Mauve alignment of the *B. coahuilensis* genomes (strains m4-4, m2-6, and p1.1.43)**. Colored rounded boxes represent syntenic regions and white/gray areas represent unique regions in the three genomes. Blue rectangular boxes indicate genomic islands that include phage-related genes. Rectangular pink boxes indicate unique areas that include genes related to transposons. Rectangular red boxes indicate unique areas that include insertion sequences. The rectangular yellow box indicates a genomic island with genes associated with the biosynthesis of cell wall lipopolysaccharide, common only in Gram-negative bacteria. The rectangular green box indicates a unique region encoding genes related to phosphonate transport in the *B. coahuilensis* m2-6 genome.

**FIGURE 4:**
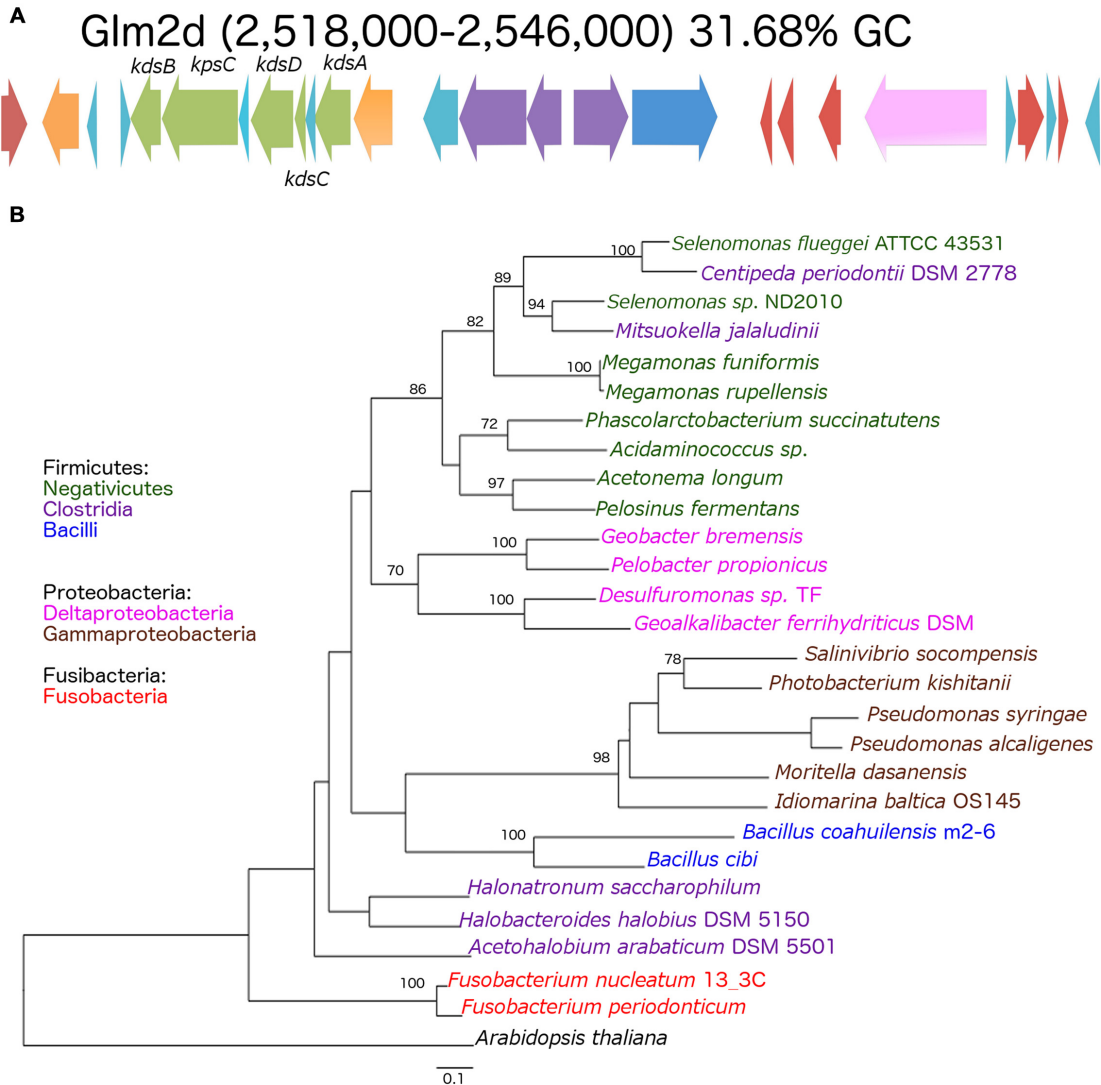
**Genomic island (GIm2d) of *Bacillus coahuilensis* m2-6 and phylogenetic reconstruction of KdsB. (A)** Genes arrangement in GIm2d. Red arrows indicate insertion sequences (IS643); orange arrows indicate genes coding for glycosyltransferase; light blue arrows indicate genes encoding hypothetical proteins; green arrows indicate genes associated with Kdo biosynthesis; purple arrows indicate genes coding for membrane proteins; the dark blue arrow indicates a gene coding for *N*-acetylmuramoyl-L-alanine amidase; the pink arrow indicates a gene coding for gamma-D-glutamyl-L-diamino acid endopeptidase I. Name of the genes associated with Kdo biosynthesis: *kdsB*, CMP-Kdo synthetase; *kpsC*, capsular polysaccharide export system protein; *kdsD*, D-arabinose-5-phosphate isomerase; *kdsC*, Kdo-8-P phosphatase; *kdsA*, Kdo-8-P synthase. **(B)** The phylogenetic reconstruction of the KdsB associated with Kdo biosynthesis was based on a maximum likelihood method. Numbers next to the branches represent bootstrap values expressed as percentages of 100 replications; only the values >70% are indicated. Bar represents 0.1 substitutions per nucleotide position. The *Arabidopsis thaliana* KdsB was used as outgroup. Colors represent bacterial lineages.

The genomes shared a high amount of genetic material and exhibited a high level of synteny. However, differences in gene content indicated that the chromosomes had suffered changes from rearrangement and HGT events. We confirmed the identity of the GI using the GIST to determine whether the unique segments present in the genomes were associated with MGEs (Hasan et al., 2012). Several unique areas (i.e., GIs) were observed in the three genomes. Two GIs associated with transposons (GIm4a and GIm4c) and other containing phage-related genes (GIm4b) were present in the m4-4 genome. Two GIs (GIp1a and GIp1b) associated with an IS, and phage-related genes, respectively, were present in strain p1.1.43. Two GIs associated with ISs (GIm2a and GIm2c) and two phage-related genes (GIm2b and GIm2e) were present in strain m2-6 (**Figure 3**). Unexpectedly, a GI was present in the m2-6 genome (GIm2d) that had a significantly lower percentage value for GC content (31.68%) than that observed for this genome (37.97%; **Figure 4A**). Analysis of this region indicated that it contained genes associated with the biosynthesis of lipopolysaccharide (LPS). We found genes for the biosynthetic pathway for saccharide 2-keto-3-deoxymannooctulosonic acid (Kdo), which is an important component of the cell wall of Gram-negative bacteria. We also found that sequences encoding several hypothetical genes, and ISs were present in this GI (**Figure 4A**). We performed a phylogenetic analysis using a sequence alignment for the KdsB protein to explore the evolutionary origin of the genes associated with Kdo biosynthesis that were identified in GIm2d of strain m2-6. The alignment of deduced amino acid sequences provided a total of 260 aligned residues, 42 of which were unambiguous. The KdsB proteins from the Fusobacteria class were a group with a high support value (100). The *kds*B gene products from the Negativicutes class group that included some of the Clostridia class also had high support values (86). Another well-supported group (70) included the KdsB proteins from the Deltaproteobacteria. The KdsB from strain m2-6 formed a well-supported group (bootstrap of 100) with *Bacillus cibi* (isolated from traditional Korean fermented seafood), and these two protein sequences were most closely related to KdsB proteins from the Gammaproteobacteria class (**Figure 4B**).

### Reduction of Amino Acid Biosynthetic Capabilities

Experiment to determine amino acid biosynthesis capabilities revealed that there were numerous auxotrophies to amino acids. Strain m2-6 had more auxotrophies (11) than strains p1.1.43 (8) and m4-4 (7). In all three strains, arginine auxotrophy was congruent with a predicted incomplete urea cycle. Valine, leucine, and isoleucine were required by the three strains. These results were consistent with the metabolic model, which revealed that the pyruvate pathway was extensively eroded. Auxotrophies that varied between strains were proline, cysteine, aspartic, threonine, and lysine. Glutamic and phenylalanine were required by all three strains. Despite this, with the metabolic models, we did not find a defective pathway to explain the auxotrophies for these seven amino acids. This result suggested that there were strain-specific regulatory differences in the expression of genes involved in the biosynthesis of amino acid. *In silico* metabolic models also revealed that strain m4-4 had an alternative pathway for the serine biosynthesis via transformation of pyruvate into serine by L-serine ammonia-lyase. An optional methionine biosynthesis pathway using a salvage pathway in which methylthioadenosine was recycled as methionine was also present (**Table 2**).

**Table 2:** Utilization of different amino acid sources by each *Bacillus coahuilensis* strain.

		Evaluation of auxotrophies			Prediction of biosynthetic pathway		
Biosynthetic pathways	Amino acid	m4-4	m2-6	p1.1.43	m4-4	m2-6	p1.1.43
a-Keto-glutarate	E	–	–	–	+	+	+
	Q	+	+	+	+	+	+
Urea cycle	P	+	–	+	+	+	+
	R	–	–	–	–^∗^	–^∗^	–^∗^
3-P-glycerate	S	+	–	–	–^∗a^	–^∗^	–^∗^
	G	+	+	+	+	+	+
	C	+	–	+	+	+	+
Oxalacetate	D	+	–	+	+	+	+
	N	+	+	+	+	+	+
	M	+	+	+	–^∗a^	–^∗a^	–^∗a^
	T	–	+	–	+	+	+
	K	+	–	+	+	+	+
Pyruvate	A	+	+	+	+	+	+
	V	–	–	–	–^∗∗^	–^∗∗^	–^∗∗^
	I	–	–	–	–^∗∗^	–^∗∗^	–^∗∗^
	L	–	–	–	–^∗∗^	–^∗∗^	–^∗∗^
Phosphoenolpyruvate and Erythrose 4-P	W	+	+	+	+	+	+
	F	–	–	–	+	+	+
	Y	+	+	+	+	+	+
Ribose 5-P	H	+	+	+	+	+	+
		
	**Total**	**7**	**11**	**7**	**6**	**6**	**6**

*Amino acids are in standard abbreviation of 1-Letter. Evaluation of auxotrophies columns: (+) Growth, (–) No Growth. Prediction of biosynthetic pathway columns: (+) complete, (–) incomplete; (^∗^) one gene is absent in pathway, (^∗∗^) more than one gene is absent, (^a^) alternative biosynthetic pathway*.

### Diversification in Carbohydrate Scavenging Capabilities

**FIGURE 5:**
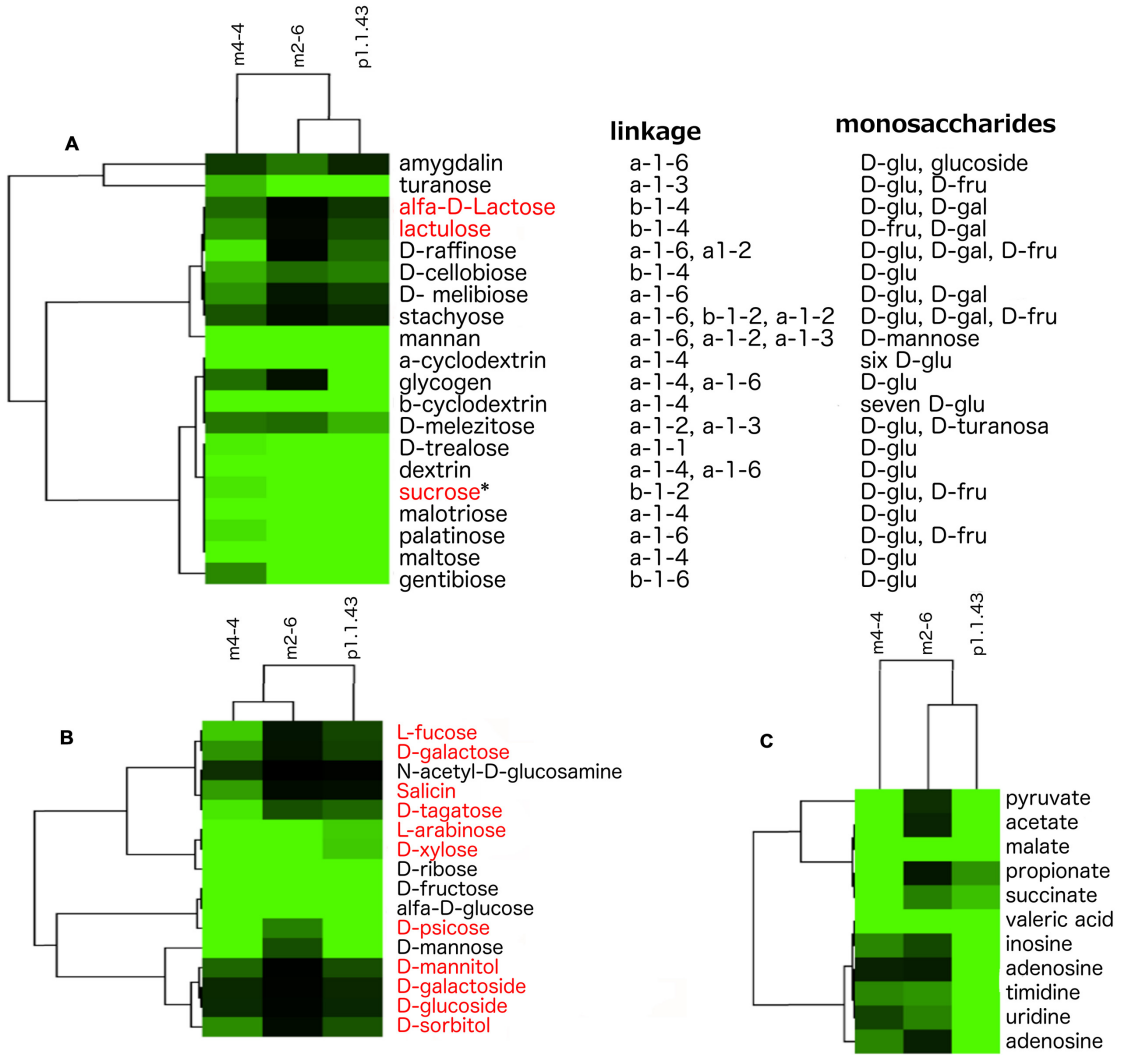
**Observed variability in the utilization of carbon sources in three *B. coahuilensis* strains. (A)** Biolog results for the use of poly- and oligosaccharides. Compounds were clustered according to how the strains (m4-4, m2-6, and p1.1.43) used poly- and oligosaccharides. **(B)** Biolog results for the use of monosaccharides. Data were clustered according to bacterial utilization, as in **(A)**. **(C)** Biolog results for the use of carboxylic acids and aromatic compounds. Data were clustered according to bacterial utilization, as in **(A)**. Carbohydrates names in red font indicate that *in silico* metabolic models have missing genes in the three genomes. ^∗^Indicates that genes are missing only in *B. coahuilensis* m4-4. In all cases, lighter green areas indicate greater evidence of source utilization, and black green indicates no utilization for poly- and oligosaccharides **(A)**, monosaccharides **(B)**, and carboxylic acids and aromatic compounds **(C)**, respectively.

Our results showed that strain p1.1.43 was more versatile in the use of carbon sources; however, all the strains used poly- and oligosaccharides of D-glucose, D-fructose, D-mannose, and D-turanose, which are mostly linked by alpha bonds. Moreover, our metabolic models suggested that genes encoding alpha- and beta-glucosidases for the hydrolysis of several di- and oligosaccharides of D-glucose and D-fructose were present, but we did not detect genes encoding galactosidase for the hydrolysis of disaccharides of D-galactose. In addition, specific transport systems for raffinose, melibiose, and stachyose were identified. However, experimental analysis using Biolog to determine the strain’s capabilities to utilize different carbon sources did not reveal evidence that any of the strains could use these saccharides (**Figure 5A**). The Biolog results and metabolic models indicated that all three strains could use the monosaccharides D-ribose, D-fructose, and alpha-D-glucose. Strain m4-4 could use a higher number of monosaccharides, compared with strains m2-6 and p1.1.43. Only strains m4-4 and m2-6 efficiently used the precursors of hemicellulose (L-arabinose and D-xylose). However, when we performed the *in silico* metabolic models, we found apparently incomplete pathways for L-arabinose, D-xylose, and for other reactions required for the synthesis of some other monosaccharides (**Figure 5B**). The Biolog results also revealed that strain m4-4 had more flexibility in the assimilation of carboxylic acids (pyruvic acid, acetic acid, L-malic acid, propionic acid, succinic acid, and valeric acid) compared with the other two strains. Strain p1.1.43 was the only strain capable of using the aromatic compounds inosine, adenosine, thymidine, uridine, and 2′-deoxy adenosine. However, results for *in silico* metabolic models suggested that all genomes had genes for membrane transport and assimilation of the carboxylic acids and the aromatic compounds tested (**Figure 5C**).

### Between-Strain Variability in Motility

**FIGURE 6:**
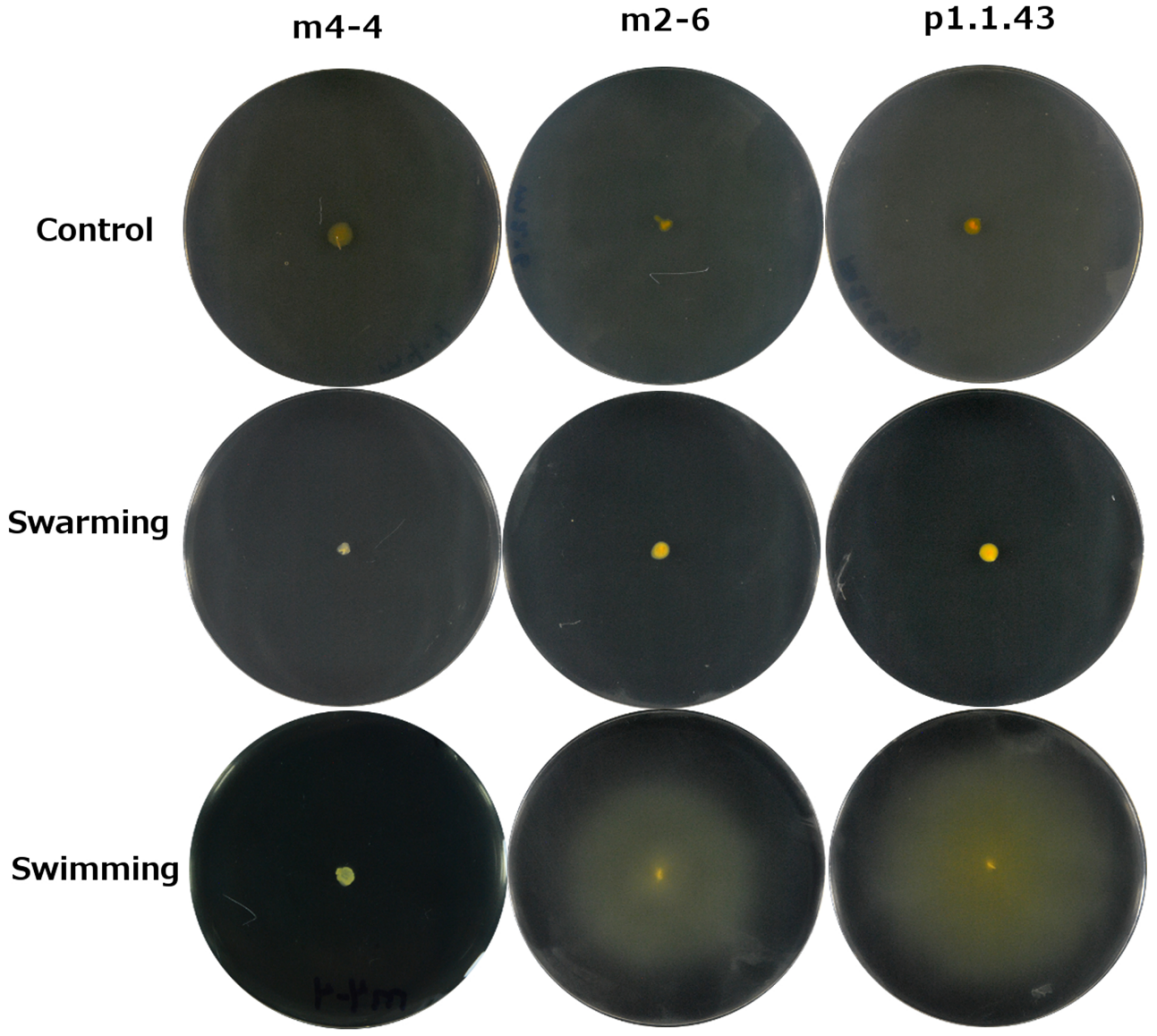
**Differences in swim capabilities and lack of swarm ability of three *B. coahuilensis* strains**. The control is marine medium (MM) containing 2% agar. Swarm was evaluated on MM containing 0.6% agar. Swim was evaluated on MM containing 0.3% agar.

Our assays revealed that strains m2-6 and p1.1.43 had the ability to swim while m4-4 lacked this ability (**Figure 6**). When we compared these results with the *in silico* metabolic model, we found that the genome of all three had the same number of genes associated with flagellar biosynthesis and bacterial chemotaxis. The results of the swarming tests were negative for all three strains (**Figure 6**). Swarming depends on the flagellar synthesis and on surfactin production. All strains lacked the *srfABCD* operon that encodes surfactin, a lipopeptide required for swarming (Supplementary Table S1).

### Different Strategies for Metabolism of Inorganic and Organic P Sources

The ability to synthesize membrane sulfolipids is used by several organisms to maintain membrane phospholipids as a P reserve (Alcaraz et al., 2008). In this sense, we found that both *B. coahuilensis* m2-6 and *B. coahuilensis* p1.1.43 genomes had sulfolipid biosynthesis genes, encoding sulfoquinovose synthase (Sqd1) and glycosyltransferase (SqdX). The experiments revealed that the membranes in all three strains had both sulfolipids and phospholipids (data not shown). We also analyzed other characteristics that could result advantageous in a low P environment, such as growth in media with different inorganic and organic P sources, and alkaline phosphatase activity. Our results indicated that the strains exhibited some differences in the assimilation of P sources. All three strains preferred to use the inorganic P source, KH_2_PO_4_. Only strain p1.1.43 used Ca_3_(PO_4_)_2_. None used phosphite (Na_2_HPO_3_; **Table 3**). On the other hand, consistent with the experimental analysis, the metabolic model analysis identified genes encoding the phosphate transport system (*pstABCS*), and the metabolism of phosphates, but not the genes *ptxABC* for phosphite transport and oxidation (Supplementary Table S2).

**Table 3:** Utilization of different phosphate sources by each *Bacillus coahuilensis* strain.

		*Bacillus coahuilensis* strains		
Type of source	Phosphorus source	m4-4	m2-6	p1.1.43
Inorganic	Na_2_HPO_3_	–	–	–
	KH_2_PO_4_	+	+	+
	Ca_3_(PO_4_)_2_	–	–	+
	X-P	–	–	–
Organic	DNA	+	+	+
	RNA	–	–	–
	2-AE-phosphonic acid	–	–	–
	Phosphonoacetaldehyde	+	+	+
	No phosphorus	–	–	–

*(+) Growth, (–) No Growth*.

Additionally, we found that all three strains are able to recycle organic phosphorus, and scavenging phosphorus from phosphonates. The results for the use of organic P sources, particularly the nucleic acids (DNA and RNA), revealed that the strains were DNA, but not RNA, as the sole source of P (**Table 3**). Our results of the metabolic model analysis indicated that the possible genes involved in the metabolism of organic P included genes for an alkaline phosphodiesterase, and a 2′, 3′- cyclic-nucleotide 2′-phosphodiesterase (**Table 4**). The results from a BLAST analysis using the SubtiList database^2^ indicated that the 2′, 3′- cyclic-nucleotide 2′-phosphodiesterase gene had an identity of 29% with gene *yfkN*, which encodes a secreted phosphodiesterase in *B. subtilis.* A third gene encoded an alkaline phosphomonoesterase (PhoA).

**Table 4:** Strategies for the recycling, scavenging, and storage of phosphorus.

Strategies	Genes	m4-4	m2-6	p1.1.43
Recycling	Alkaline phosphodiesterase I	+	+	+
	2′, 3′ cyclic nucleotide transferase (*yfkN*)	+	+	+
	Alkaline phosphatase (*phoA*)	Constitutive	–	Induce
Scavenging	Phosphonoacetaldehyde dehydrogenase (*phnY*)	+	+	+
	Phn transporters (*phnCDE1E2*)	–	+	–
Storage	Polyphosphate kinase *ppK*	+	+	+
	Exopolyphosphatase	+	+	+
	Teichoic acids biosynthesis genes	–	–	–
	*sqd1*	+	+	+
	*sqdX*	+	+	+

*Constitutive and induce refers to *phoA* gene expression*.

Some bacteria can use C-P containing organic P sources (e.g., phosphonates). All three strains were able to grow in the presence of phosphonoacetaldehyde, but not in the presence of 2-aminoethylphosphonic acid (**Table 3**). A comparison of these experimental results with the *in silico* metabolic model indicated that there were no genes associated with phosphonate utilization. We developed hidden Markov models for different enzymes associated with phosphonate metabolism. We identified in all three genomes a gene encoding an aldehyde dehydrogenase that matched to a phosphonoacetaldehyde dehydrogenase gene (*phnY*), with a significant *E*-value (1*e*-^111^ to 3.8 *e*-^111^; Supplementary Table S2). This enzyme (PhnY) catalyzes the transformation of phosphonoacetaldehyde to phosphonoacetate (Villarreal-Chiu et al., 2012). In addition, a gene cluster associated with phosphonate transport that was present in strain m2-6 was absent in the other two genomes. We also searched for genes related to phosphorus storage (biosynthesis of polyphosphate and teichoic acids). We observed in all three genomes the genes coding for the synthesis and hydrolysis of polyphosphates, but not genes related to teichoic acids biosynthesis (**Table 4**).

**FIGURE 7:**
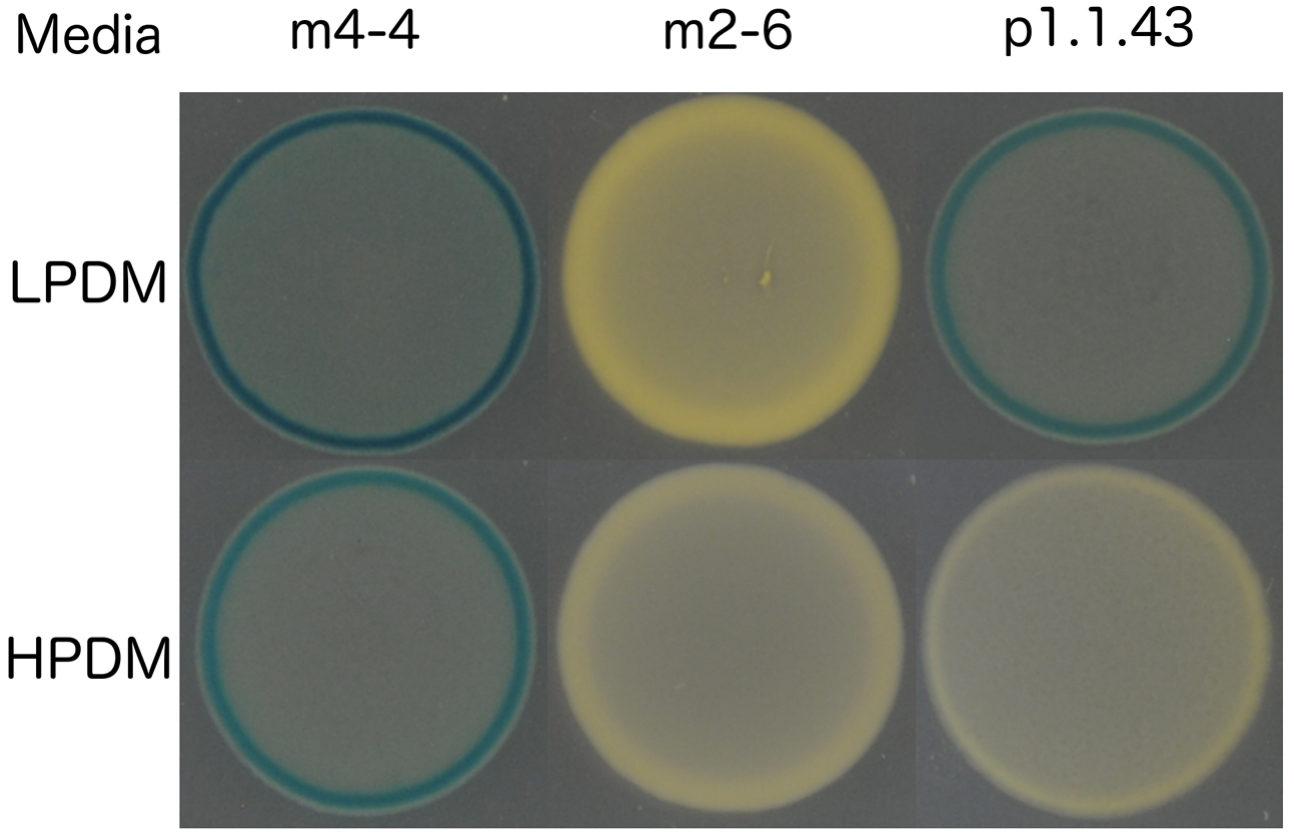
**Intraspecific differences in alkaline phosphatase activity in three *B. coahuilensis* strains (m4-4, m2-6, and p1.1.43)**. LPDM, low phosphate defined medium; HPDM, high phosphate defined medium with KH_2_PO_4_. X-Pi, 5-Bromo-4-chloro-3-indolyl phosphate (40 μM/mL) was used as an indicator of alkaline phosphatase activity.

The three strains exhibited differences in the presence of the alkaline phosphate gene and its regulation. Consistent with this result was the alkaline phosphatase activity assay that showed constitutive alkaline phosphatase activity for strain m4-4 (expressed both in high and low KH_2_PO_4_ concentration). Alkaline phosphatase activity also occurred in strain p1.1.43, but this activity was induced only in low phosphate concentration conditions. No activity was observed for *B. coahuilensis* strain m2-6 (**Figure 7**). These results suggested that microevolutionary changes had affected phosphorus utilization strategies, both through gene loss (e.g., the alkaline phosphatase gene in m2-6) and through changes in gene regulation (**Table 4**).

### HGT Origin of Phosphonate Transport in *B. coahuilensis* m2-6

**FIGURE 8:**
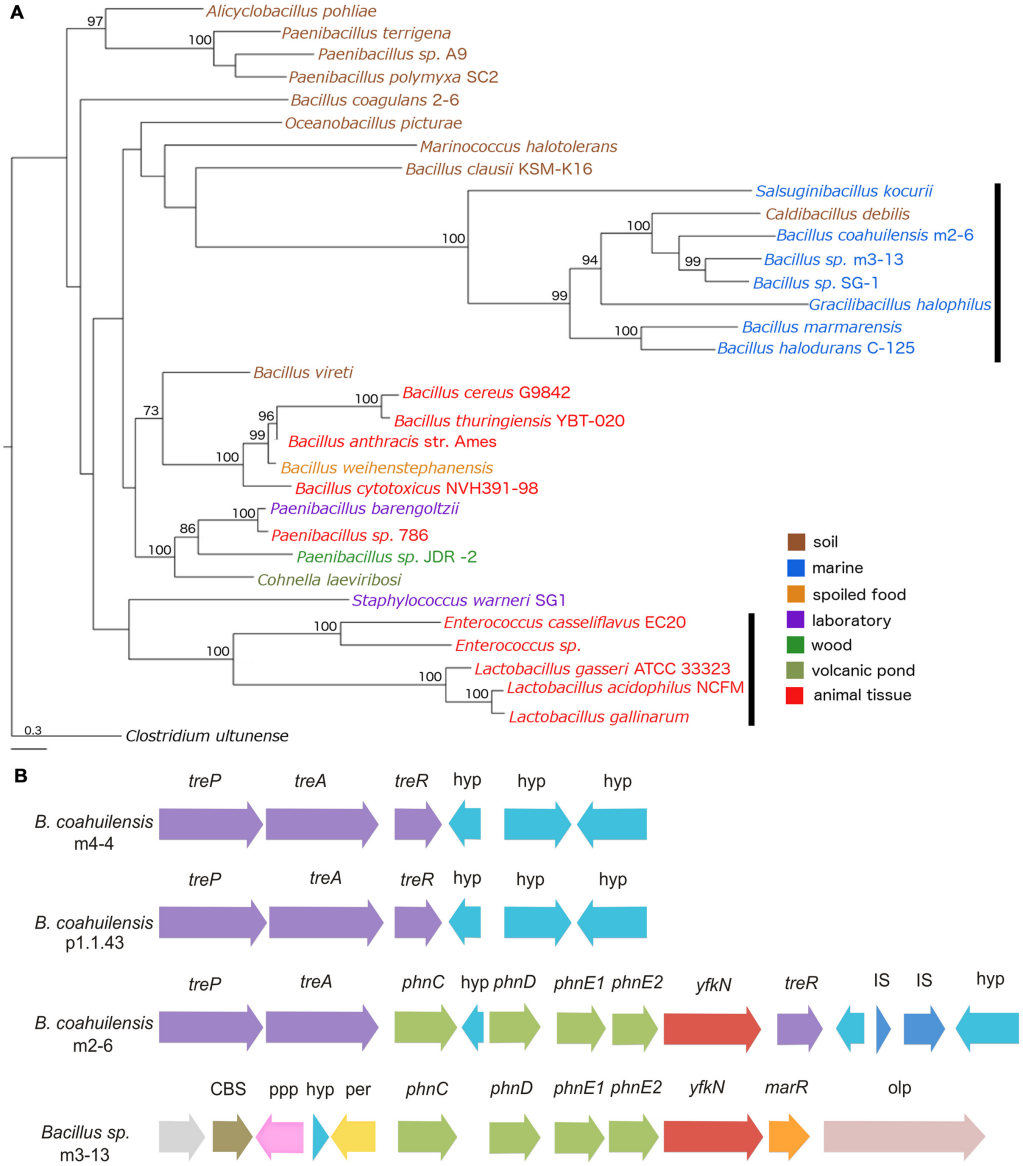
**Phylogeny of Phn transport cluster and genomic context identified in *B. coahuilensis* strain m2-6. (A)** Phylogenetic tree of concatenated Phn sequences (PhnC, PhnD, PhnE1, and PhnE2) was performed with a Maximum Likelihood method. Numbers next to the branches represent bootstrap values expressed as percentages of 100 replications; only values >70% are indicated. Bar represents 0.3 substitutions per nucleotide position. *Clostridium ultunense* was used as outgroup. Colors represent bacterial isolation environments. The black vertical bars indicate events of HGT, as suggested for *Lactobacillus* and *Enterococcus* (Huang et al., 2005). **(B)** Genomic neighborhood of phosphonate transport cluster (*phnC, phnD, phnE1*, and *phnE2*) in *B. coahuilensis* m2-6. *treP*, PTS system trehalose-specific IIB component; *treA*, trehalose-6-phosphate hydrolase; *treR*, trehalose operon transcriptional repressor; hyp, hypothetical protein; *yfkN*, 2′ 3′-cyclic-nucleotide 2′-phosphodiesterase; IS, insertion sequences; CBS, cystathionine beta-synthase domain protein; ppp, putative periplasmic protein; *per*, zinc/iron permease; *marR*, transcriptional regulator of MarR family; *olp*, oligopeptidase F.

To explore the possible evolutionary origin of the phosphonate (*phn*) transport cluster identified in *B. coahuilensis* m2-6, we performed a phylogenetic analysis using the multiple protein sequence alignments for the *phn* transport genes (*phnC, phnD, phnE1*, and *phnE2*). The concatenated alignments of the deduced amino acid sequences provided a total of 1159 aligned residues, of which 425 were unambiguous. The Phn transporters from members of the *Bacillus cereus* group form another well-supported clade. The Phn gene products from the *Alicyclobacillus pohliae* were associated with some species of the genus *Paenibacillus* with high support (>90). Another well-supported group (100) included lineages of *phn* genes from *Enterococcus* and *Lactobacillus* species. The Phn transporter from strain m2-6 formed a well-supported group (100) with other *Bacillus* species isolated from aquatic environments, and with *Salsuginibacillus kocurii*, *Caldibacillus debilis*, and *Gracilibacillus halophilus* (**Figure 8A**). The presence of some well-supported monophyletic groups formed by different bacterial lineages suggested that several HGT events occurred in the class Bacilli (**Figure 8A**). The comparison of the phylogeny of Phn transporter with the species phylogeny (16S rRNA; Supplementary Figure S1) indicated discordances between Phn transporter and species tree that can be suggestive of an HGT event. Finally, to understand the genomic context and the biological role of the *phn* transport cluster in strain m2-6, we analyzed the genomic neighborhood of the *phn* transport cluster. We found that it was located within the trehalose operon (*treP*, *treA*, and *treR*). The results of a comparison of the genomic neighborhood of the *phn* transport cluster of strain m2-6 with *Bacillus* sp. m3-13 (isolated from the Churince system in the CCB) indicated that both gene clusters maintained a similar organization, and each had a gene coding for a 2′ 3′-cyclic-nucleotide 2′-phosphodiesterase (**Figure 8B**). The results of a BLAST analysis using the SubtiList database indicated that this gene was highly similar to *B. subtilis yfkN*, which encodes a secreted phosphodiesterase (27% identity). The biological role of this enzyme in the metabolism of phosphonates by *B. coahuilensis* is unknown.

## Discussion

### Genomic Diversity and Variation Associated to MGEs

Comparative genomics is a valuable approach to reveal in great detail the genetic variation among bacteria at species, strain, and individual levels, as well as to answer evolutionary questions on gene loss, gain, and transfer. In the present study, we performed a phenotypic-genotypic association analysis of the *B. coahuilensis* strains to understand the genomic and metabolic diversity. We wanted to explore the differences between strains with a relatively small genome, and exposed to similar oligotrophic conditions. We explored their ability to use different carbon sources, *in silico* and using Biolog and, since the strains were isolated from the same low P environment we emphasized the search of different strategies for phosphorus transport, metabolism, storage and regulation.

We observed high ANI values between *B. coahuilensis* genomes (98.8 and higher), which reflects the genetic distance. These highly ANI values have been observed in other organisms that have an ecological constrains (Konstantinidis et al., 2006), and is consistent with the oligotrophic environment from which the strains were isolated. The core genome and pangenome was informative of the potential gene pool available to *B. coahuilensis*. Overall, the three *B. coahuilensis* genomes shared 82% of their genes. This result was consistent with other intraspecific comparative genomics studies (Deng et al., 2010; Peña et al., 2010; Mann et al., 2013; Patiño-Navarrete et al., 2013). Most of the strain-specific genes encoded hypothetical genes and 5 to 8% of their genes encoded MGEs, mainly related to phages, CRISPS, transposons, and ISs. Our results agree with studies of other bacterial lineages, which found that the genomes are shaped by the presence of MGEs (Peña et al., 2010; Kalhoefer et al., 2011; Meyer and Huber, 2014). On the other hand, the alignment of the three *B. coahuilensis* genomes exhibited high synteny, probably as a consequence of very few genomic rearrangements. This result was consistent with the results of comparative genomic analysis of co-occurring environmental *Lebetimonas acidiphila* strains (Meyer and Huber, 2014). Also in genome alignment, we observed different GIs, in which the strain-specific genes were concentrated. The GIm2d found just in strain m2-6 encoded genes involved in the biosynthesis of Kdo, an important component of the LPS of the cell wall of Gram-negative bacteria. The phylogenetic reconstruction of KdsB from strain m2-6 indicated that the genes for Kdo biosynthesis were acquired by HGT. The acquisition of novel functional genes via HGT increases the potential for specific adaptations and microbial speciation in the CCB. This is an example of a microevolutionary change in this species, and it is also an unexpected finding for the Firmicutes that is worth describing. It will be interesting to elucidate the function of this cell-surface component.

### Metabolic Analysis Uncovers that *B. coahuilensis* is Highly Dependent on Nutrients and a Diversification in Carbohydrate Scavenging Capabilities

The phenotypic and genotypic analysis revealed that the *B. coahuilensis* strains had lost numerous capabilities related to amino acid biosynthesis. This species probably is highly dependent on nutrients available in the sediment microbial community. The loss of some biosynthetic pathways in all three strain genomes may be due to a genome-size reduction, and/or an adaptation to a unique particular niche (Alcaraz et al., 2008), which can be referred as streamlining, a term used to describe gene loss occurring in bacteria (Giovannoni et al., 2005, 2014). It has been suggested that gene loss occurs particularly in microbial communities, as the result of changes of availability of public goods (Morris et al., 2012).

However, we found that some auxotrophies detected experimentally could not be explained by gene loss as the *in silico* metabolic models revealed complete amino acid biosynthetic pathways. Other phenotypic-genotypic association inconsistencies observed were related to swimming ability in strain m4-4, supporting the idea that regulation in gene expression also has contributed to the intraspecific diversity of *B. coahuilensis.* Therefore, we suggest that gene loss is probably not the only strategy to achieve energy efficiency because it implies a high risk, given the lack of environmental stability for free-living bacteria. Changes in gene expression allow efficient energy utilization, and also to conserve genetic information. Similar inconsistencies between *in silico* metabolic models and auxotrophies have been observed in other species of *Bacillus* genus isolated from CCB hydrological systems (data not shown). Nevertheless, these phenotypic-genotypic inconsistencies support the idea that the magnitude of the differences between genomes cannot be inferred merely from genomic comparison. Phenotypic data brings the true dimension of a cell’s function. Metabolic models performed so far require feedback from experimental data, to connect genome predictions to phenotypes. The main difficulty of metabolic predictions is precisely the near impossibility to include predicted regulatory features. Regulatory information gathered from the literature is crucial to improve metabolic models. We believe that in all organisms under study, the evolution through changes in gene regulation is yet under-appreciated given the need to uncover it experimentally. Understand the phenotype to genotype correlation is one of the most important issues of the post genomic era.

On the other hand, the genotypic-phenotypic association was more consistent for carbohydrate utilization, where most of the metabolic models agreed with the results of carbohydrate utilization obtained through Biolog analysis. However, we found that some pathways (L-arabinose and D-xylose) could not be fully elucidated, suggesting that unlike other model bacteria, *B. coahuilensis* may use alternative enzymes. Similar results have also been obtained in studies of other non-model bacteria such as *Roseobacter litoralis* (Kalhoefer et al., 2011). Based on the results of the utilization of alternative C sources (e.g., carboxylic acids and aromatic compounds), we suggest that there has been a diversification in carbohydrate scavenging capabilities among the *B. coahuilensis* strains.

### Differences in Phosphorus Acquisition Strategies and Their Regulation

Strategies to survival in a low P environment are key in the CCB, as revealed by previous analysis of the *B. coahuilensis* m4-4 genome (Alcaraz et al., 2008). Our results showed similarities and differences in the abilities of the three *B. coahuilensis* strains for phosphorus recycling, scavenging, and storage. The first similarity among all three strains was the ability to use DNA as a phosphorus source, consistent with the occurrence of genes coding for an alkaline phosphodiesterase I, and the 2′ 3′-cyclic-nucleotide 2′-phosphodiesterase, YfkN. Both enzymes are associated with the recycling of phosphorus from extracellular RNA and DNA, and are induced in response to phosphorus starvation (Vershinina and Znamenskaya, 2002; Singh et al., 2015). Another similarity was that all three genomes encoded a glycerolphosphodiesterase (GlpQ), which is an important enzyme that hydrolyzes deacylated phospholipids to glycerol-3-phosphate. In *B. subtilis*, this phosphodiesterase is strongly induced by phosphorus starvation and is regulated by the PhoP–PhoR system (Antelmann et al., 2000). The third similarity is the ability to scavenge phosphorus from phosphonate. The three genomes have the gene encoded a putative phosphonoacetaldehyde dehydrogenase (PhnY). This enzyme has been described in *Sinorhizobium meliloti* 1021 as part of a novel pathway for degradation of 2-aminoethylphosphonate, catalyzed transformation of phosphonoacetaldehyde to phosphonoacetate (Borisova et al., 2011). A fourth similarity is the presences of genes that encode polyphosphates biosynthesis. A recent study in Sargasso Sea plankton indicated that the enrichment in polyphosphate coincided with enhanced alkaline phosphatase activity and substitution of sulfolipids for phospholipids, which are both indicators of phosphorus stress (Martin et al., 2014). A fifth coincidence is the absence of the genes related to teichoic acids biosynthesis. These polymers of the cell walls of Gram-positive bacteria are considered as phosphorus storage molecules, given that they have repeating polyol or glycosylpolyol residues linked by phosphodiester bonds (Kulakovskaya, 2014). It is likely that *B. coahuilensis* lost the capacity to produce teichoic acid because of the P-limited environment of CCB (Alcaraz et al., 2008).

As for the differences, *B. coahuilensis* p1.1.43 was the only strain with the ability to use Ca_3_(PO_4_)_2_. The results of many studies have indicated that the ability to use this mineralized phosphorus source is accompanied by the ability to solubilize it by secreting organic acids (e.g., succinate, fumarate, gluconic, and malate; Deubel and Merbach, 2005; Khan et al., 2009; Sharma et al., 2013). Other between-strain differences in the P recycling, and was related to gene content, and expression of the gene *phoA* encoding alkaline phosphomonoesterase. Finally, the last difference was related to the presence of a phosphonate (*phn*) transport cluster in the strain m2-6 genome. Phosphonates have an increasingly acknowledged role in phosphorus cycling in nature. Biosynthetic and metabolic pathways that include phosphonates have been discovered (Quinn et al., 2007). The ability of microbes to cleave the C–P bond confers organisms the possibility to use a wide range of phosphonates as nutrients. The phosphonates scavenging as P reservoir in the oceans has been well established as well as the strategies that marine microorganisms employ for their utilization (Villarreal-Chiu et al., 2012). Furthermore, alternative phosphonate utilization pathways were recently described in representatives of Proteobacteria, Planctomycetes, and Cyanobacteria, commonly found in marine plankton (Martinez et al., 2010).

The genomic neighborhood analysis and comparison with the *phn* transport cluster of the *Bacillus* sp. m3-13 genome revealed variation in structure and gene composition. Unexpectedly, we also found a gene, *yfkN*, which was consistently associated with the *phn* gene cluster in the *B. coahuilensis* m2-6 and the *Bacillus* sp. m3-13 genomes. In *B. subtilis*, this gene is induced in response to P starvation. The gene product exhibits 2′, 3′ cyclic nucleotide phosphodiesterase, 2′ (or 3′) nucleotidase, and 5′ nucleotidase activities to provide pathways for the recycling of P from extracellular DNA and RNA (Allenby et al., 2005). A recent study revealed that in *Rhizobium leguminosarum*, an enzyme that originally was reported to have a phosphodiesterase function could also hydrolyze phosphonate monoesters (Jonas et al., 2008). The observation of this dual function suggested that this gene could have phosphonatase activity in strain m2-6.

Recycling phosphorus from DNA, scavenging from phosphonates, and phosphate storage are possibly acquisition strategies that, along with the ability to synthesize membrane sulfolipids, help *B. coahuilensis* to survive in a harsh environment. The evolutionary force that could explain the diversification in the phosphorus acquisition strategies could be genetic drift. Sometimes restricted dispersal creates geographically structured subpopulations (each with a reduced *N*_e_), promoting their genetic diversification (Luo and Moran, 2015). In this sense, only three strains of *B. coahuilensis* have been recovered in the last 8 years of extensive isolation efforts of *Bacillus* sp., suggesting that this species might be structured in small subpopulation with a restricted dispersal that could promote microevolutionary change. Finally, most comparative genomic studies of bacteria focus on pathogens and symbionts of humans, animals, and plants. The determination of the genomic and metabolic diversity of free-living bacteria such as *B. coahuilensis* represents an effort to understand what drives bacterial microevolution and adaptation in natural oligotrophic environments. Further transcriptomic and proteomic studies could help to reconcile the differences observed between the genotypic and phenotypic results. Another avenue open for research is the cross metabolic regulation between strains co-occurring in microbial communities.

## Conclusion

Our results revealed that an important source of the *B. coahuilensis* strains’ genome variation is associated with their numerous MGEs. We also identified the loss of some amino acid biosynthetic pathways, variability in motility ability, and a diversification in carbohydrate and phosphorus scavenging capabilities, and suggest that this microevolutionary changes result not just from gene loss and/or gain, but also from gene regulation affecting specific pathways. Particularly, we found some phosphorus acquisition strategies that enable the *B. coahuilensis* strains to recycle phosphorus, scavenging, and phosphate storage, all important strategies for survival in an environment with low concentration of this essential element.

## Author Contributions

ZG-L contributed to the conception and design of the study, to data acquisition and analysis, interpretation of the results, and preparation of the manuscript; IH-G and M-DR-T contributed to data acquisition and analysis, and to interpretation of the results; GO-A participated in the conception and design of the study, and to manuscript preparation; VS participated in a critical revision of the manuscript.

## Conflict of Interest Statement

The authors declare that the research was conducted in the absence of any commercial or financial relationships that could be construed as a potential conflict of interest.

## Footnotes

1 http://www.ncbi.nlm.nih.gov/genbank/

2 http://genolist.pasteur.fr/SubtiList/
