# Supplementary material for: Microevolution Analysis of Bacillus coahuilensis Unveils Differences in Phosphorus Acquisition Strategies and Their Regulation
Source: Front Microbiol. 2016 Feb 8;7:58. doi: 10.3389/fmicb.2016.00058 (PMC4744853; doi:10.3389/fmicb.2016.00058)
Supplement: Supplementary file 1 [file Presentation_1.PDF]

Supplementary Material

Microevolution analysis of *Bacillus coahuilensis* unveils differences in phosphorus acquisition strategies and their regulation

Zulema Gómez-Lunar[1], Ismael Hernández-González[1], María-Dolores Rodríguez-Torres[1], Valeria Souza[2], Gabriela Olmedo-Álvarez[1*]

***Correspondance:** Gabriela Olmedo Álvarez: golmedo@ira.cinvestav.mx

1 Supplementary Tables

Supplementary Table 1. Genes in the *Bacillus coahuilensis* genomes associated with flagellar biosynthesis

		Bacillus coahuilensis		
**Function**	**Role**	**m4-4**	**m2-6**	**p1.1.43**
Experimental data	Swarming	-	-	-
	Swimming	+	+	+
Surfactin synthesis	SrfABCD	-	-	-
Bacterial Chemotaxis	Inhibitor of MCP methylation CheC	+	+	+
	CheV	+	+	+
	Positive regulator CheW	++	++	++
	Signal transduction histidine kinase CheA	+	+	+
	Maltose/maltodextrin ABC transporter MalE	+	+	+
	Galactose/methyl galactoside ABC transport system MglB	+	+	+
	Response regulator protein-glutamate methylesterase CheB	+	+	+
	CheX	+	+	+
	Serine chemoreceptor protein	+++	++	++
	Methyltransferase CheR	+	++	++
	CheD	+	+	+
	Dipeptide-binding ABC transporter	+	+	+
	Two-component response regulator for flagellar switch bias CheY	++	++	++
Flagellar ATPase complex	Assembly protein FliH	+	+	+
	Specific ATP synthase FliI	+	+	+
	Protein for formation of basal body FliJ	+	+	+
Flagellar	Biosynthesis protein FlhA	+	+	+

Motor switch	Biosynthesis protein FlhB	+	+	+
	Motor switch protein FliG	+	+	+
	Motor switch protein FliM	+	+	+
	Motor switch protein FliN	+	+	+
	Biosynthesis protein FliP	+	+	+
	Biosynthesis protein FliQ	+	+	+
	Biosynthesis protein FliR	+	+	+
In cytoplasmic membrane	Motor rotation protein MotA	+	+	+
	Motor rotation protein MotB	+	+	+
	M-ring protein FliF	+	+	+
Flagellar Basal body proximal rod	Hook-basal body complex protein FliE	+	+	+
	Basal-body rod protein FlgB	+	+	+
	Basal-body rod protein FlgC	+	+	+
	Basal-body rod protein FlgF	+	+	+
Hook	Hook-length control protein FliK	+	+	+
	Basal-body rod modification protein FlgD	+	+	+
Hook-filament junction	Hook-associated protein FlgK	+	+	+
	Hook-associated protein FlgL	+	+	+
Filament	Hook-associated protein FliD	+	+	+
	Flagellin protein FlaA	++	++	+
Regulation of biosynthesis	Cell division protein FtsI	+++	+++	+++
	Synthesis regulator FleN	+	+	+
	RNA polymerase sigma factor RpoD	+	+	+
	Transcriptional activator FlbB	+	+	+
	RNA polymerase sigma-54 factor RpoN	+	++	++
	Sigma54 transcriptional activator FlbD	+	+	+
	RNA polymerase sigma factor for operon sigD	+	+	+
	Assembly factor FliW	+	+	+
	Protein FlaG	+	+	+
	Protein yvyF	+	+	+
	Transcriptional regulator of post-exponential-phase genes flaD or sinD	++	++	++
	Biosynthesis protein FlhF	+	+	+
	Biosynthesis protein FliS	+	+	+
	Protein FliT	+	+	+
	Biosynthesis protein FliZ	+	+	+
	Negative regulator of flagellin synthesis FlgM	+	+	+

(+) Indicated one gene coding, (++) indicated two genes coding and (+++) indicated three genes coding.

Supplementary Table 2. Genes in the *Bacillus coahuilensis* genomes associated with phosphorus transport, metabolism, storage and regulation

**Function**	**Subfunction**	**Role**	**Genes identified in *B. coahuilensis* genomes**		
			**m4-4**	**m2-6**	**p1.1.43**
Membrane Transport	Phosphate	PstABCS	+	+	+
	Phosphite	PtxABC	-	-	-
	Phosphonate	PhnBCE1E2	-	+	-
Metabolism	Phosphite oxidation	Phosphorus lyase	-	-	-
		PtxD	-	-	-
	Glycerol-3-phosphate uptake	GlpQ	+	+	+
	Phosphate hydrolysis	Alkaline phosphatase PhoA	+	-	+
	DNA and RNA hydrolysis	Alkaline phosphodiesterase I	+	+	+
		2', 3'-cyclic-nucleotide 2'-phosphodiesterase YfkN	+	+	+
	Phosphonates hydrolysis	Phosphono-acetaldehyde dehydrogenase PhnY	+	+	+
		Phosphonate lyase	-	-	-
	Polyphosphate hydrolysis	Exopolyphos-phatase	+	+	+
	Polyphosphate synthesis	Polyphosphate kinase PpK	+	+	+
Phosphorus storage and saving	Membrane reserve	Sqd1	+	+	+
		SqdX	+	+	+
	Wall reserve	Teichoic acids biosynthesis TagOAB/TagDEFGH	-	-	-
PHO regulon control	PhoP–PhoR system	Regulatory protein PhoU	+	+	+
		Sensor protein PhoR	+	+	+
		Transcriptional regulatory protein PhoP	+	+	+

	PhoH	++	++	++
Others	ResD	+	+	+
	ResE	+	+	+
	Spo0A	+	+	+

(+) Indicated one gene coding, (++) indicated two genes coding.

2 Supplementary Figure

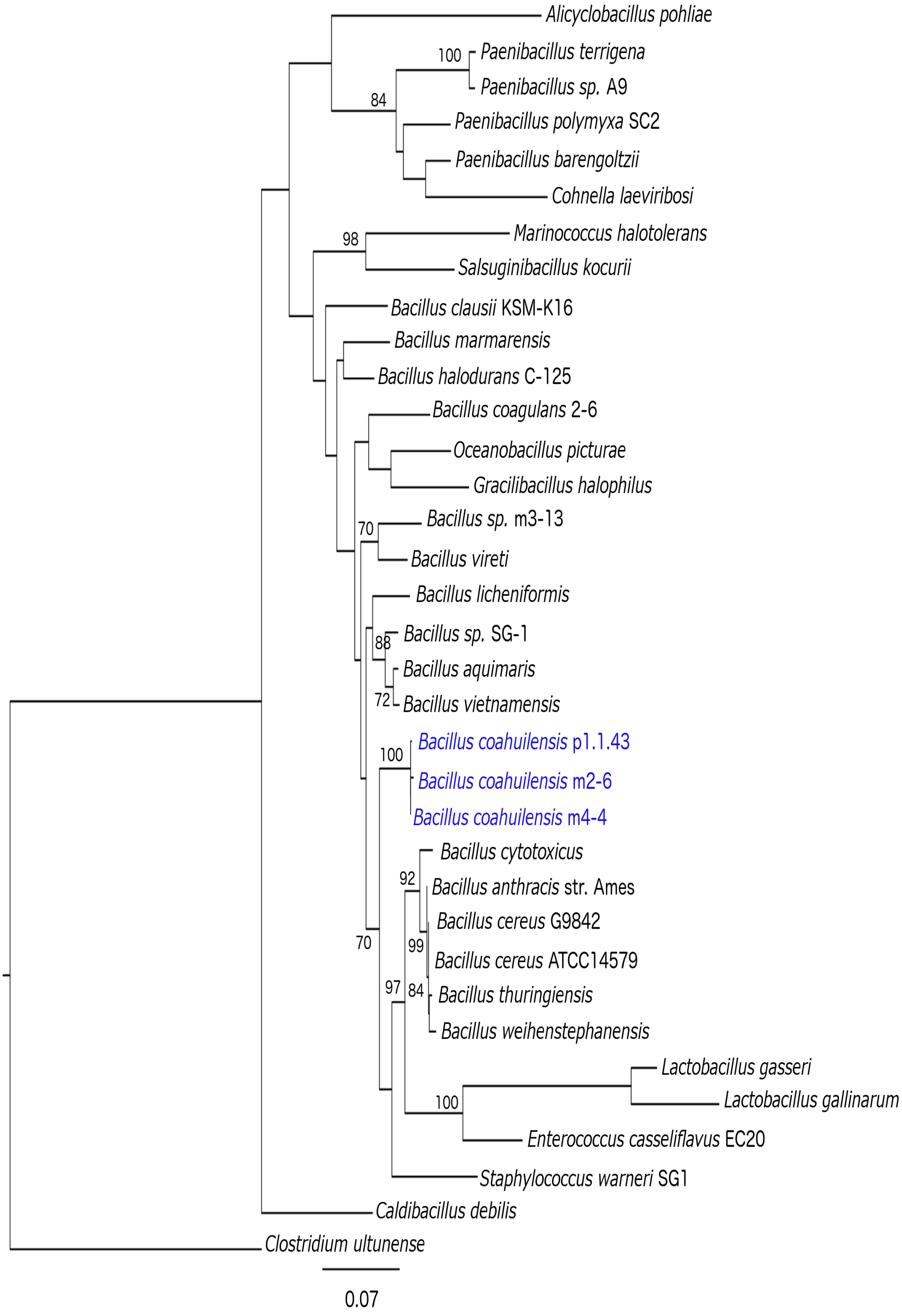

Figure 1. The phylogenetic reconstruction of the 16S rRNA was based on a maximum likelihood method. Numbers next to the branches represent bootstrap values expressed as percentages of 100 replications; only the values >70% are indicated. Bar represents 0.07 substitutions per nucleotide position. The *Clostridium ultunense*16S rRNA was used as outgroup. Blue colors represent *Bacillus coahuilensis* strains.
